# Prognostic nutritional index and the prognosis of diffuse large b-cell lymphoma: a meta-analysis

**DOI:** 10.1186/s12935-020-01535-x

**Published:** 2020-09-15

**Authors:** Chunyan Luan, Fei Wang, Ning Wei, Baoan Chen

**Affiliations:** 1 Medical School of Southeast University, Nanjing, 210009 China; 2 Department of Hematology and Oncology (Key Department of Jiangsu Medicine), Southeast University Affiliated Zhongda Hospital, No. 87 Dingjiaqiao, Nanjing, 210009 China; 3 Department of Gastroenterology, Southeast University Affiliated Zhongda Hospital, Nanjing, 210009 China

**Keywords:** Meta-analysis, Prognostic nutritional index, Diffuse large B-cell lymphoma, Prognosis

## Abstract

**Background:**

Some studies have investigated the prognostic value exhibited by the Prognostic Nutritional Index (PNI) in patients suffering diffuse large B-cell lymphoma (DLBCL), but varying results were obtained. In order to determine the specific prognostic value more accurately, a meta-analysis was conducted in this study.

**Methods:**

Literatures were searched from the China National Knowledge Infrastructure (CNKI), Wanfang, PubMed, Embase, the Cochrane Library, and Web of Science. Pooled hazard ratio (HR) and the 95% confidence interval (CI) were calculated to assess the association between PNI and the overall survival (OS) and the progression-free survival (PFS) of patients with DLBCL.

**Results:**

Based on seven studies with a total number of 1311 patients, our meta-analysis revealed that low PNI may meant poor OS (HR = 2.14, 95% CI 1.66–2.75, p < 0.001) and poor PFS (HR = 1.75, 95% CI 1.36–2.25, p = 0.438). Subgroup analysis showed that, in Asians, low PNI was correlated to poor OS (pooled HR = 2.06 95% CI 1.59–2.66) and poor PFS (pooled HR = 1.66, 95% CI 1.28–2.15). Similar results were obtained from one European study, which is the only study performed outside of Asia from our literature search.

**Conclusion:**

For patients with DLBCL, low PNI may be interpreted as adverse prognosis. More data from European patients are required in this study to avoid analysis bias.

## Introduction

As the most commonly diagnosed tumor in adults, diffuse large B-cell lymphoma (DLBCL) constitutes about 20% of newly diagnosed lymphoid neoplasms [1]. In Western countries, DLBCL accounts for 31% of all non-Hodgkin’s lymphomas (NHL) [2]. Due to the biological and clinical heterogeneity of the tumor, DLBCL patients are typically treated strategically with different drugs, such as rituximab, cyclophosphamide, vincristine, doxorubicin, and prednisone; R-CHOP [3]. Although about 60%-70% of patients suffering DLBCL are curable by different regimens, chemotherapy is insensitive for some patients who sometimes exhibit a poor long-term survival outcome [4]. Gene expression profiling (GEP), International Prognostic Index (IPI) and other indexes are useful for identifying high-risk patients [5, 6], however they are not easily available in daily clinical practice and are incapable of predicting prognosis accurately. Therefore, there is an urgent call for the development of simple and easily accessible prognostic biomarkers at a low cost.

A number of studies in recent years have shown that malnutrition, which is a frequently-encountered issue in patients with DLBCL, is associated with the poor overall survival (OS) [7–9]. Lymphoma patients with poor nutrition supply have a higher risk of developing febrile neutropenia that can lead to delays in chemotherapy treatment due to decreased drug usage. Recent studies have found that PNI, an indicator that reflects the nutritional and immune status of patients, can be used to predict the clinical outcomes of patients with various malignant tumors, regardless of the tumor location and origin [10–14]. Some studies have focused on exploring the prognostic value of PNI for DLBCL, however the results were inconsistent and contradictory [3, 15–20], possibly due to small sample sizes and patient heterogeneity in individual studies. In order to achieve a comprehensive evaluation of PNI for DLBCL, we aggregated the data from related studies and performed a meta-analysis to investigate how PNI is used in predicting the OS and the progression-free survival (PFS) of patients.

## Materials and methods

### Search strategy

Literatures published since inception till April 2020 from PubMed, Embase, Cochrane Library, Web of Science, CNKI (Chinese), and Wanfang were searched using search terms (“Prognostic Nutritional Index”) AND (Lymphoma), and evaluated by two investigators (N.W. and CY.L) independently. A consensus was reached to resolve conflicting opinions during the searching process. Relevant studies referenced in the literatures were also examined.

### Selection criteria

Literatures with the following features were included in our meta-analysis: (1) DLBCL patients must be diagnosed by histology; (2) Must contain prognostic value of PNI for OS and/or PFS, or with sufficient data for relevant calculation; (3) Hazard Ratio (HR) must be reported as the prognostic index (4) The PNI must be calculated before the first chemotherapy cycle. Meanwhile, articles in the form of comments, reviews, case reports, or thesis were excluded from our study. Latest articles with the largest sample size were chosen in our analysis.

### Data extraction and quality assessment

The data were extracted independently by two investigators (N.W and CY.L). A third investigator (BA.C) participated in discussions to resolve discrepancies. Date of eligible studies including author, country, publication year, sample size, patient age, treatment plans, DLLBCL state, cut-off values exhibited by PNI, follow-up time, and survival outcomes were extracted. The quality of the included studies was assessed based on the Newcastle–Ottawa Scale (NOS) [21], where studies with a score of ≥ 6 out of 9 were regarded as high quality research.

### Statistical analysis

HR as well as the 95% confidence interval (CI) values were pooled using Stata version 15.0 (STATA, College Station, TX) to evaluate the association of different level of PNI and OS and PFS. All HR and 95%CI were extracted directly from the included articles. Heterogeneity of the included studies was evaluated by Q and I^2^ statistics. Random effects model was used when the data were considered highly inconsistent with I^2^ > 50% or P < 0.05; otherwise, fixed effect model was used instead. For sensitivity analysis, the result credibility of each study was examined by sequential omission. Publication bias was evaluated using Egger’s test by STATA. A two-tailed p-value of < 0.05 was considered statistically significant.

## Results

### Search results

**Fig. 1:**
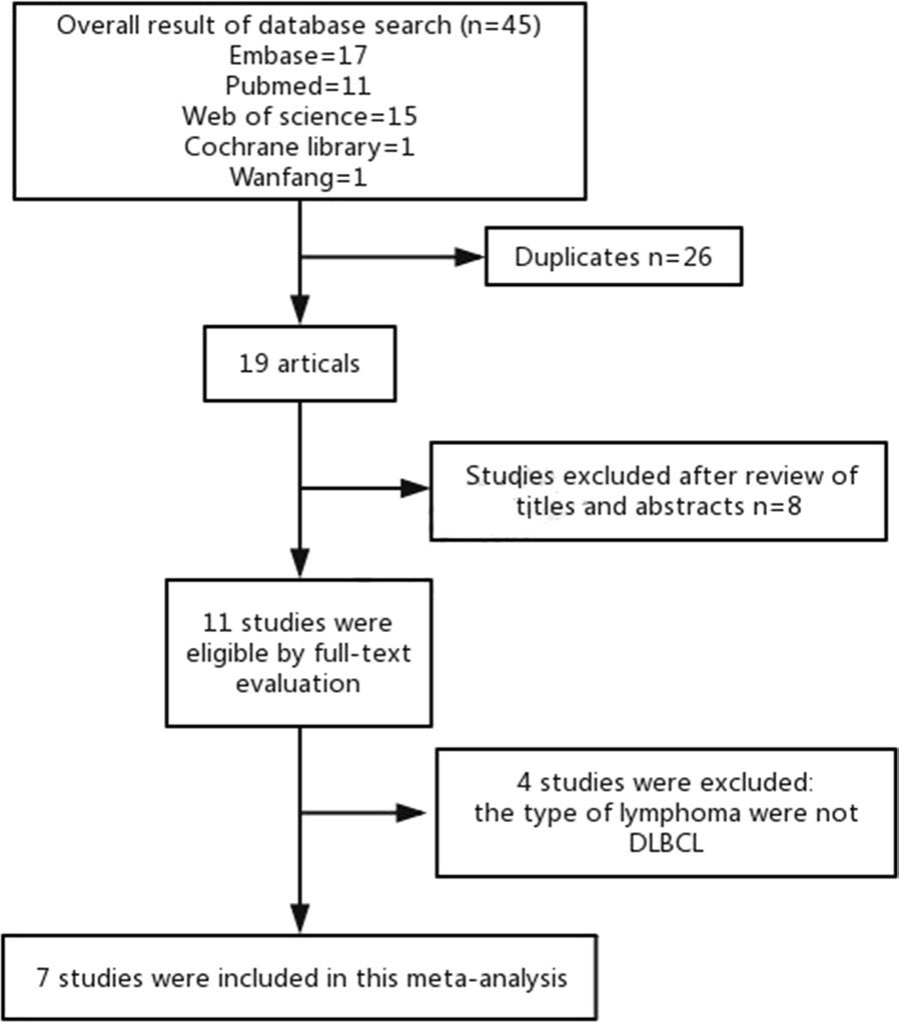
Flow chart of the screening process in choosing eligible studies

Initial literature search included a total of 45 studies, but only 19 studies remained following the removal of duplicating studies. Another 8 studies were excluded after title/abstract screening. Due to the type of lymphoma not being DLBCL, 4 full-text articles were further removed. As a result, a total of 7 relevant studies were included in the current meta-analysis [15−20, 22] (Fig. 1).

### Features of included studies

Out of the 7 chosen studies that were published from 2016 to 2020, 4 were conducted in China [15−17, 19], while the remaining 3 were each carried out in Korea [18], Japan [20] and Croatia [22], respectively. In terms of language, 6 studies were published in English [15, 16, 18–20, 22] and 1 in Chinese [17]. The total sample size was 1311, and the cut-off values of PNI were between 40 and 45. Whilst R-CHOP or R-CHOP like regimen was employed in 3 studies [18, 19, 22]; R-CHOP/CHOP or CHOP like regimen was used in another 3 studies [15–17]; whereas rituximab-containing chemotherapy regimens (R-CHOP /R-CVP/rituximab alone) and palliative therapy were used in the remaining study [20]. The prognostic values of PNI on OS [15−20, 22] were reported in all 7 studies; while the association between PNI and PFS [15, 16, 18, 22] was shown in 4 studies. All included studies had a NOS score of ≥ 6 (Table 1).

**Table 1:** Features of the studies included

Author	Country	Year	Sample size (high/low PNI)	Cut-off value of PNI	Median (range) of PNI	Adjusted factors	Follow-up time (month)	Age (year)(range)	NOS score	Treatment	Stage	Survival outcome
Xiaoxiao Hao	China	2017	125/127	45	–	IPI, GPS, NLR, PNI, PI	–	49 (16–82)	6	R-CHOP; CHOP/CHOP-like	I–IV	OS, PFS
Wenjuan Yu	China	2019	114/195	45	48.4 (23.9–86.2)	BMI, hemoglobin, NCCI-IPI	–	–	7	R-CHOP	I–IV	OS
Se-Il Go	Korea	2019	69/159	40	–	Sarcopenia, albumin, ALC, BMI, IPI, B-symptoms	–	64 (21–88)	7	R-CHOP	I–IV	OS, PFS
Vlatka Periˇsa	Croatia	2017	75/28	44.55	50.26 (22.91–65.3)	Age, gender, IPI ECOG-PS, LDH, Ann Arbor stage, B-symptoms	Median: 27 (range: 1–105)	63 (22–87)	6	R-CHOP/ R-CHOP-like	I–IV	OS, PFS
Qinjun Zhou	China	2016	129/124	44.675	-	B-symptoms, LDH, Ann Arbor stage, ECOG-PS, extra-nodal, IPI	–	49 (19–81)	6	R‑CHOP	I–IV	OS, PFS
Teng Song	China	2019	44/38	44.15	–	ECOG-PS, Ann Arbor stage, LDH,IPI, ALC	–	59(23–79)	6	CHOP; R-CHOP	I–IV	OS
Erina Hamada	Japan	2020	38/46	41.3	–	Albumin, ALC, IPI, extra-nodal ECOG-PS, LDH, gender, Ann Arbor stage, B-symptoms	Median: 39	84 (80–94)	6	R-CHOP; R-CVP; R alone; palliative	I–IV	OS

*IPI* International Prognostic Index, *PNI* Platelet Lymphocyte Ratio, *PNI* Prognostic Nutritional Index, *GPS* Glasgow prognostic score, *PI* Prognostic Index, *NLR* Neutrophil Lymphocyte Ratio, *R-CHOP* rituximab plus cyclophosphamide doxorubicin vincristine and prednisone, *BMI* Body Mass Index, *ECOG PS* Eastern Cooperative Oncology Group performance status, *LDH* lactate dehydrogenase, PFS progression-free survival, *OS* overall survival, *ALC* Absolute lymphocyte count, *NCCN* National Comprehensive Cancer Network

### Results of meta-analysis

**Fig. 2:**
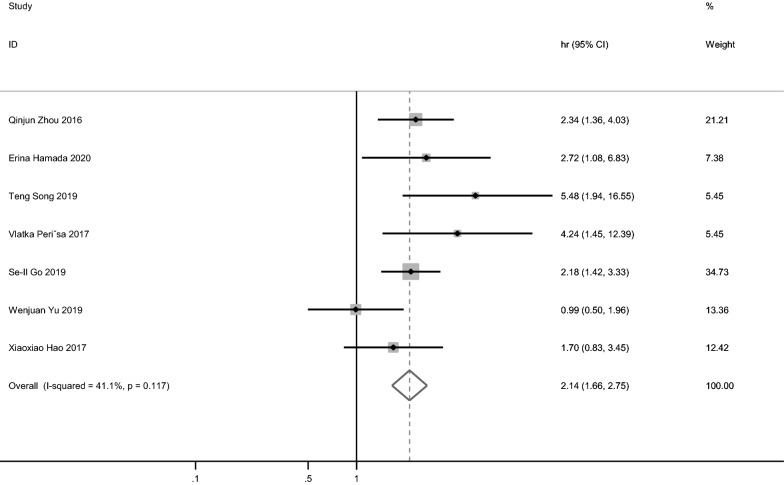
Pooled results of the association between PNI and overall survival (OS)

All 7 studies [15−20, 22] reported the correlation between PNI and OS. Fixed-effect model was used with P = 0.117 and I^2^ = 41.1%. Our meta-analysis showed that a low PNI was significantly correlated to worse OS (HR = 2.14, 95% CI 1.66–2.75; Fig. 2, Table 2). Due to the lack of obvious heterogeneity, only ethnic (Asian or not) subgroup analysis was conducted to study the impact of PNI on OS. The combined results of six studies indicated that PNI was still a significant marker in Asian (Pooled HR = 2.06. 95% CI 1.59–2.66); while the only study with non-Asian showed that PNI also had a significant predictive value (HR = 4.24, 95% CI 1.451–12.392).

**Table 2:** Results of subgroup meta-analysis

Group	No. of studies	HR (95% CI)	Heterogeneity	
			I^2^ (%)	P
OS	7	2.14(1.66–2.75)	41.1	0.117
Ethnicity				
Asian	6	2.06(1.59–2.66)	41.4	0.129
Non-Asian	1	4.24(1.451–12.392)	–	–
Other treatment	4	2.43(1.68–3.51)	8.2	0.352
PFS	4	1.7 (1.36–2.25)	39.2	0.117
Ethnicity				
Asian	3	1.66(1.28–2.15)	4.7	0.350
Non-Asian	1	4.007(1.48–10.852)	–	–

**Fig. 3:**
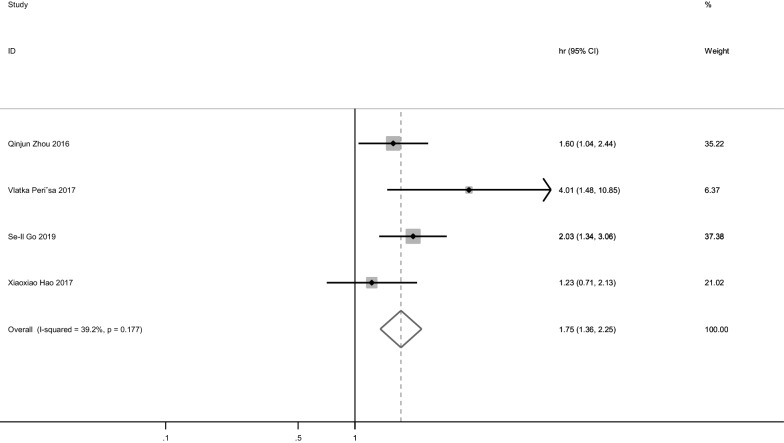
Pooled results of the association between PNI and progression-free survival (PFS)

The association of PNI and PFS was reported in four studies [15, 16, 18, 22], which included 836 patients. Due to the low heterogeneity, a fixed-effect model was applied (P = 0.177, I^2^ = 39.2%). Our analysis showed that the pooled HR was 1.75 with a 95% CI of 1.36–2.25 (Table 2, Fig. 3), indicating that lower PNI and poorer PFS are closely associated. Subgroup analysis revealed that PNI was correlated to PFS of Asian (pooled HR = 1.66, 95% CI 1.28–2.15). Meanwhile, the only European study suggested that PNI can predict PFS (HR = 4.007 95% CI 1.48–10.852).

### Sensitivity analysis and publication bias evaluation

**Fig. 4:**
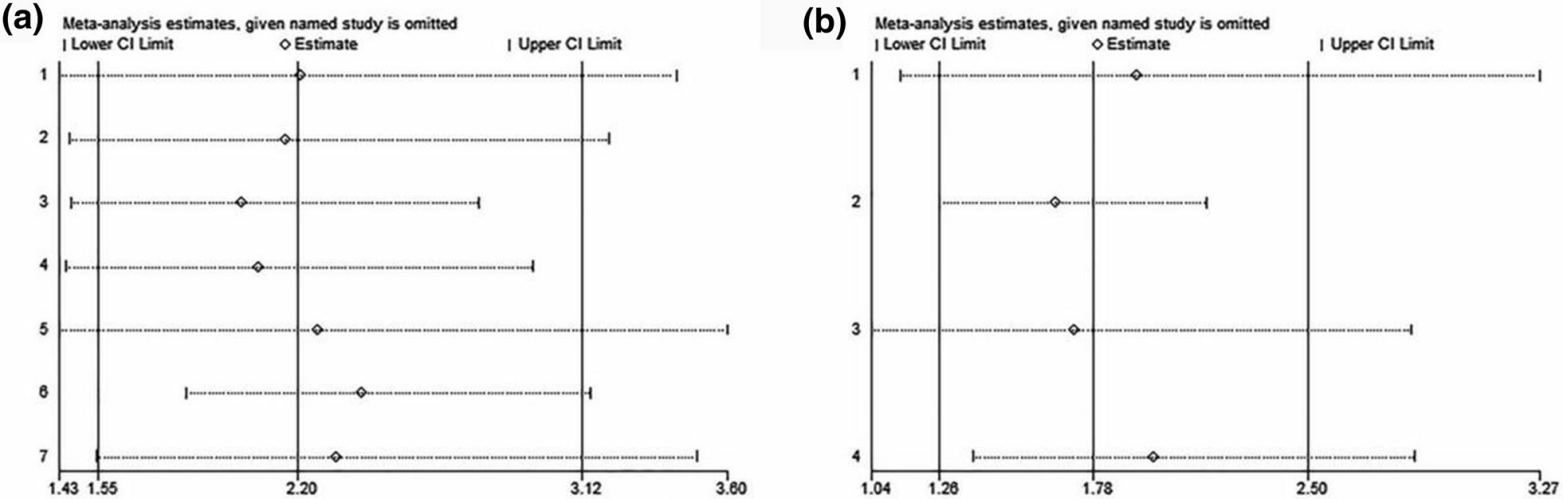
Sensitivity analysis of the pooled hazard ratios (HRs) to evaluate the association between PNI and OS (**a**) and PFS (**b**)

Sensitivity analysis showed that changes of the pooled HRs of OS or PFS remain insignificant following omission any individual study (Fig. 4), indicating that the results were reliable.

**Fig. 5:**
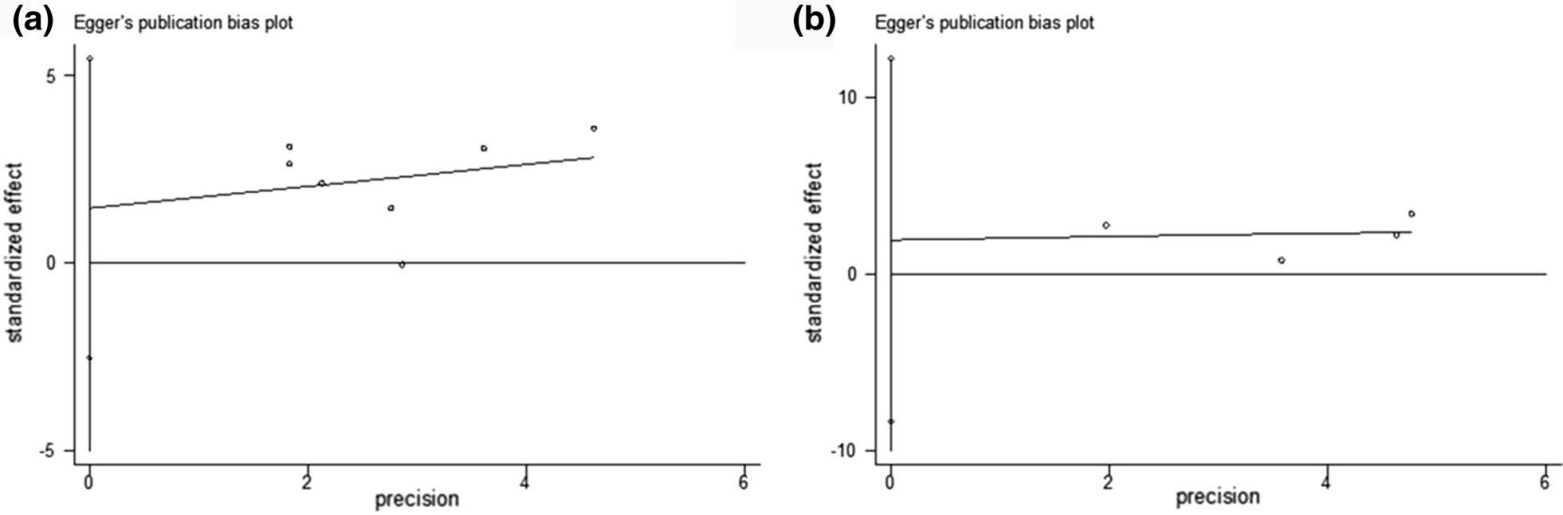
Publication bias analysis using Egger’s test for OS (**a**) and PFS (**b**)

No publication bias was found in the this meta-analysis (Egger’s test: OS, p = 0.391; PFS, p = 0.509) (Fig. 5).

## Discussion

Tumor progression has been shown to be remarkably affected by inflammation and nutrition [23]. Recent studies have identified a simple prognostic score based on nutritional status and PNI as biomarkers that can be used to independently predict the prognosis of DLBCL patients in terms of OS and PFS. PNI was initially used for assessing patients receiving digestive tract surgery due to immunological and nutritional complications [24-27]. Later, it was found that PNI could simply be used to powerfully predict the prognosis of various diseases, including solid tumors and hematological diseases. Previous studies have revealed that PNI exhibit a prognostic value for DLBCL patients [15−20, 22]. However, whilst most studies [15, 17, 18, 20, 22] have demonstrated PNI as a significant prognostic factor for DLBCL patients; two studies have reported the opposite results [16, 19].

To our knowledge, our meta-analysis was the first study that focused on the prognostic value exhibited by PNI in DLBCL patients. In this study, data aggregation was performed from 7 studies that covered 1311 patients in total. Our results showed that, regardless of ethnicity, low PNI was a significant prognostic marker for poorer OS (pooled HR = 2.14, 95% CI 1.66–2.75) and poorer PFS (pooled HR = 1.7 95% CI 1.36–2.25).

Although the accurate mechanism of how low PNI is associated with poor prognosis remains unclear, there are a number of possible explanations: 1) hypoalbuminemia may be due to malnutrition, and malnourished patients may show a worse response to treatments as well as a weaker treatment tolerance compared to that of well-nourished patients; (2) the decreased concentrations of serum albumin and lymphopenia were possibly due to cytokine release by the tumors, such as tumor necrosis factor-alpha and interleukin 6, indicating that the disease is strongly aggressive; (3) low ALC caused by the pre-existing immunosuppression, indicating that the antitumor immunological reaction of the host was insufficient; (4) low ALC that was possibly caused by lympholytic cytokines arising from lymphoma cells, and this kind of lymphomas could exhibit an intrinsic treatment resistance [28-32].

## Limitation

There were several limitations identified in our study. Firstly, our analysis involved a relatively small sample size, including only 7 studies where most of the data were obtained from Asian countries. Accordingly, the predictive value of PNI in European countries requires further discussion. In addition, our analysis only involved studies published in Chinese and English, excluding those that were reported in other languages.

## Conclusion

Low PNI may represent adverse prognosis in patients with DLBCL. However, since our analysis mainly focused on Asian studies, our findings should be interpreted with caution in European patients.

## Data Availability

The databases analyzed during the current study are available.
